# Novel magnetic propylsulfonic acid-anchored isocyanurate-based periodic mesoporous organosilica (Iron oxide@PMO-ICS-PrSO_3_H) as a highly efficient and reusable nanoreactor for the sustainable synthesis of imidazopyrimidine derivatives

**DOI:** 10.1038/s41598-020-67592-4

**Published:** 2020-06-30

**Authors:** Arezoo Akbari, Mohammad G. Dekamin, Amene Yaghoubi, Mohammad Reza Naimi-Jamal

**Affiliations:** Pharmaceutical and Heterocyclic Compounds Research Laboratory, Department of Chemistry, Iran University of Science and Technology, Tehran, 1684613114 Iran

**Keywords:** Environmental sciences, Chemistry, Materials science, Nanoscience and technology

## Abstract

In this study, preparation and characterization of a new magnetic propylsulfonic acid-anchored isocyanurate bridging periodic mesoporous organosilica (Iron oxide@PMO-ICS-PrSO_3_H) is described. The iron oxide@PMO-ICS-PrSO_3_H nanomaterials were characterized by Fourier transform infrared spectroscopy, energy-dispersive X-ray spectroscopy and field emission scanning electron microscopy as well as thermogravimetric analysis, N_2_ adsorption–desorption isotherms and vibrating sample magnetometer techniques. Indeed, the new obtained materials are the first example of the magnetic thermally stable isocyanurate-based mesoporous organosilica solid acid. Furthermore, the catalytic activity of the Iron oxide@PMO-ICS-PrSO_3_H nanomaterials, as a novel and highly efficient recoverable nanoreactor, was investigated for the sustainable heteroannulation synthesis of imidazopyrimidine derivatives through the Traube–Schwarz multicomponent reaction of 2-aminobenzoimidazole, C‒H acids and diverse aromatic aldehydes. The advantages of this green protocol are low catalyst loading, high to quantitative yields, short reaction times and the catalyst recyclability for at least four consecutive runs.

## Introduction

The use of heterogeneous catalysts has been developed because of their desirable properties and addressing many principles of green chemistry. Therefore, development and research in the heterogeneous catalysts has received major consideration due to disadvantages associated with homogeneous catalysts such as catalyst recovery, product separation, corrosion problems and environmental hazards^1–5^. Along these lines, development of nanoporous materials with significant improved properties is a new and growing research field in the recent years^6–10^. Highly-ordered periodic mesoporous organosilicas (PMOs) materials, as a kind of inorganic-organic hybrid mesoporous materials, have attracted significant interest because of their noteworthy properties such as high surface area, narrow pore size distribution, adjustable mesopore diameter, high mechanical and hydrothermal stability, and highly tunable physicochemical properties by varying the nature and extent of the surface functionalization.

PMOs which are mainly prepared from bridged organosilane precursors [(R′O)_3_Si-R-Si(OR′)_3_; R: organic bridging group, R′: methyl or ethyl] have found vast applications in various fields such as drug delivery systems, absorption and storage of mechanical energy, gas storage, electronics, sensors, luminescence, adsorbents, chromatography, solid-phase extraction, etc. Also, these type of materials have been used as suitable supports in a diversity of heterogeneous catalysts for different organic reactions^11–29^. Indeed, the mesopore channels and high surface area of PMOs make them as an appropriate nanoreactor for releasing of the reactants into mesoporous channels and increases the reaction rate^30–41^. In addition to the above mentioned properties, another special features of PMOs are uniform distribution of active organic groups within their framework to tune their polarity compared to nanoordered silica materials and reusability^13^. Among the various organic groups used in the PMOs structure, the heteroaromatic isocyanurate ring containing three nonpolar alkyl groups shows excellent properties including nontoxicity, highly branched, binding ability to transition metals and high thermal stability^14,34,35,42–47^. On the other hand, the inclusion of magnetic nanoparticles (MNPs) in the modified materials allows convenient and cost-effective separation to be conveniently performed by an external magnetic field instead of centrifugation and filtration steps. Furthermore, MNPs enhance the reaction rates by local heating through induction as well as providing appropriate surface area. Also, they show synergistic effects in combination to other catalytic species or centers, due to the catalytic performance of magnetic materials, including Fe, Ni, or Co-based ones^27,48–54^. Therefore, the synergistic effects of both PMO-based organosilicas and magnetic components for designing and application of new materials would be very desirable. To the best of our knowledge, a little efforts have been made for designing of magneic PMO materials^54–56^, especially thermally stable isocyanurate-based mesoporous organosilica solid acid which are in high demand for promoting of organic reactions at elevated temperatures.

**Figure 1:**
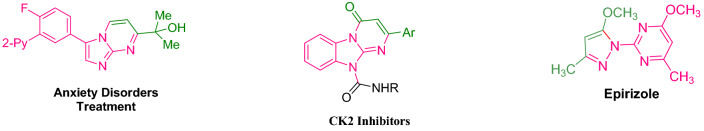
Representative examples of biologically active imidazopyrimidine derivatives.

On the other hand, development of simple synthetic procedures for the synthesis of complex and diversity-oriented organic molecules from readily available substrates is an important challenge in organic and medicinal chemistry. This can be achieved through multicomponent reactions (MCRs) strategy as a powerful process for the synthesis of molecules useful for pharmaceuticals, biological studies, secret communication and electronic including heterocyclic scaffolds^57–62^ as well as fabrication of new task-specific materials such as drug delivery systems, nanocomposites, polymers, supramolecular systems and molecular machines^63–68^. In MCRs, three or more reactants simultaneously combine together in one reaction vessel to form a final product with high bond forming index such as imidazopyrimidine derivatives^69–77^. Indeed, imidazopyrimidine derivatives show a diverse range of biological and pharmacological activities such as CK2 inhibitor as well as for the treatment of anxiety disorders and ulcers, etc. (Fig. 1)^78–82^.

**Scheme 1:**
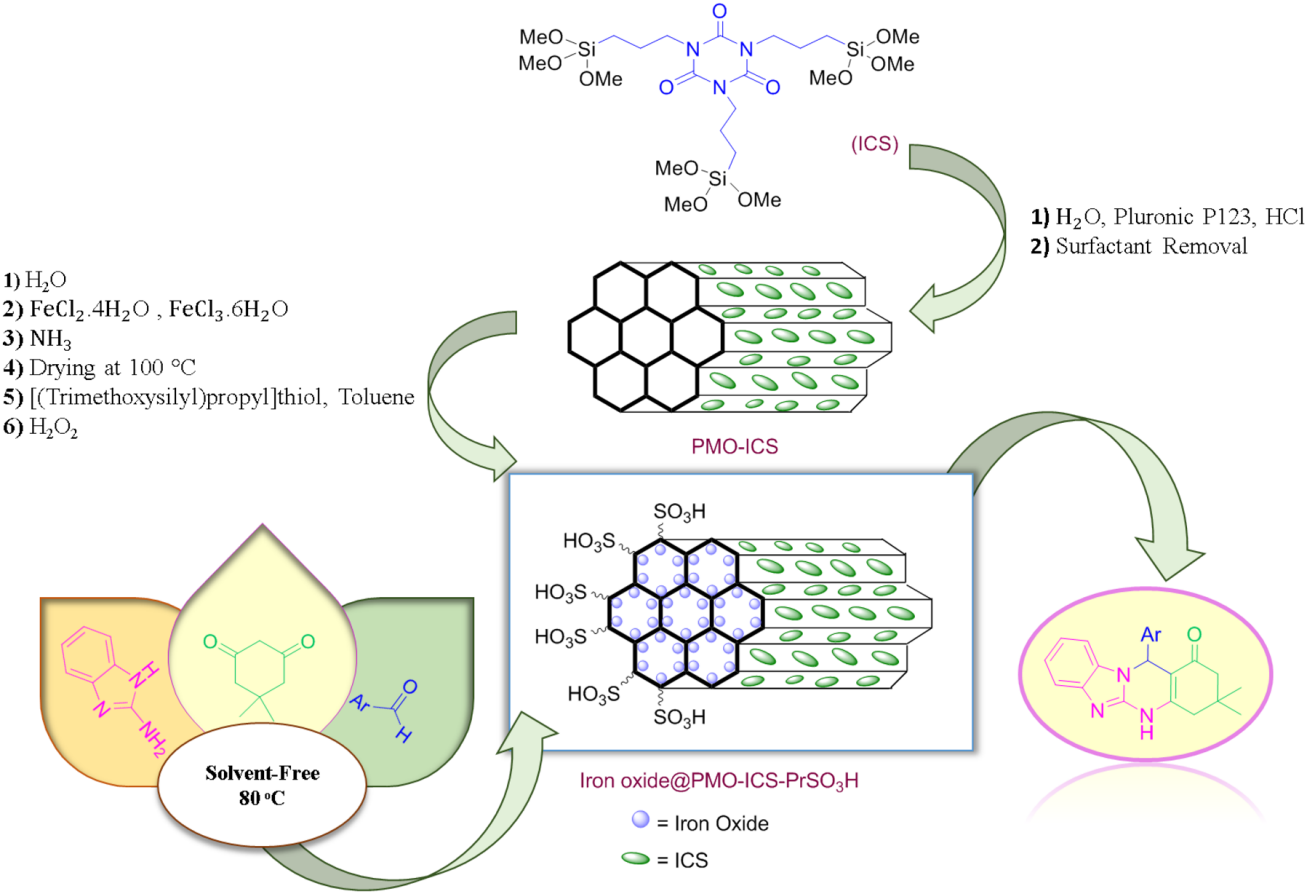
Schematic preparation of the Iron oxide@PMO–ICS–PrSO_3_H nanocatalyst (**1**) and its application in the synthesis of imidazopyrimidine derivatives **6a–g** and **7a–g**.

Because of the importance of imidazopyrimidine scaffold, different homogeneous or heterogeneous acidic catalytic systems have been investigated to promote multicomponent condensation of 2-aminobenzoimidazole, aromatic aldehydes and C–H acids such as dimedone/malononitrile or relevant synthons. Some of the recent examples of reported catalysts are H_6_P_2_W_18_O_62_.18H_2_O^83^, WO_3_-supported sulfonic acid^84^, O-sulfonated poly(vinylpyrrolidonium) hydrogen sulfate^85^, modified ZnO nanoparticles under ball milling conditions^86^, organo-sulfonic acid tags anchored on magnetic titana coated NiFe_2_O_4_ nanoparticles^87^, Fe_3_O_4_@GO^88^, magnetic Irish moss^80^, carboxymethyl cellulose^89^, NH_2_SO_3_H^90^, *p-*toluenesulfonic acid monohydrate^91,92^, Fe_3_O_4_@clay^93^, L-proline^94^, molecular iodine^95^, polyethylene glycol methacrylate-grafted dicationic imidazolium-based ionic liquid^96^ and NaHSO_4_ modified phenylene bridged periodic mesoporous organosilica magnetic nanoparticles^55^. In spite of their merits, the existing methodologies have drawbacks such as low to moderate yields, difficulties in the catalyst recovery and product isolation, toxic or expensive catalysts, lengthy reaction times, the use of volatile organic solvents or significant amounts of waste materials production^97^. Therefore, development of new methodologies and introducing green catalysts to overcome the aforementioned drawbacks is still favorable. To address limitations and disadvantages associated with these catalytic systems, preparation and catalytic application of magnetic isocyanurate-based propylsulfonic acid periodic mesoporous organosilica (Iron oxide@PMO-ICS-PrSO_3_H), as a novel and highly efficient heterogeneous mesoporous catalyst, would be very desirable. In continuation of our research interest to develop and improve novel and efficient catalysts for different MCRs or organic transformations^34,35,51,52,73,74,89,98,99^, we wish herein to report the application of Iron oxide@PMO-ICS-PrSO_3_H (**1**), as a novel recyclable catalyst, for the synthesis of imidazopyrimidine derivatives through the Traube–Schwarz multicomponent reaction under solvent-free conditions. To the best of our knowledge, there is no report on the use of Iron oxide@PMO-ICS-PrSO_3_H, as a nanao-architectured heterogeneous and recoverable catalyst, for different organic transformations (Scheme 1).

## Results and discussion

### Characterization of the Iron oxide@PMO-ICS-PrSO_3_H nanomaterials (1)

**Figure 2:**
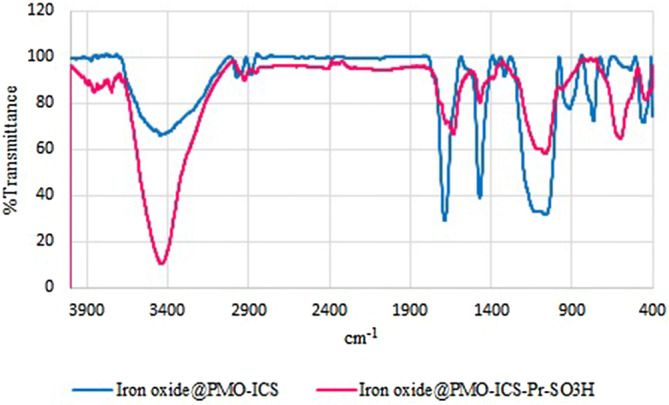
FT-IR spectra of PMO-ICS mesoporous catalyst (blue spectrum) and Iron oxide@PMO-ICS-PrSO_3_H magnetic mesoporous catalyst (**1**, pink spectrum).

After preparation of the magnetic isocyanurate-based propylsulfonic acid periodic mesoporous organosilica (Iron oxide@PMO-ICS-PrSO_3_H) nanocatalyst (**1**), its composition, structure, morphology and textural properties was properly characterized by different methods and techniques. The FT‐IR spectra of both magnetic Iron oxide@PMO-ICS (B) and Iron oxide@PMO-ICS-PrSO_3_H (**1**) nanomaterials have been compared in Fig. 2. As it can be seen in Fig. 2, the absorbance bands at 2,925 and 2,857 cm^−1^ are related to C–H stretching of the aliphatic moiety in the catalyst **1** or its precursor nanomaterials B. Furthermore, absorption bands in the regions 1,120, 1,070 and 933 cm^−1^ correspond to the asymmetric and symmetric vibrations of Si–O–Si (siloxane) vibrations, respectively. Moreover, the signals appeared at 1633 and 1,470 cm^−1^ are attributed to the stretching vibrations of the isocyanurate ring. Also, the absorption bands at 1,284–1,177 and 1,134–1,045 as well as 630–572 cm^−1^ were assigned to the O = S = O asymmetric or symmetric and S–O stretching vibration of the –SO_3_H functional group, respectively. Furthermore, the band observed at 480 cm^−1^ could be attributed to the Fe–O spinel structure.

**Figure 3:**
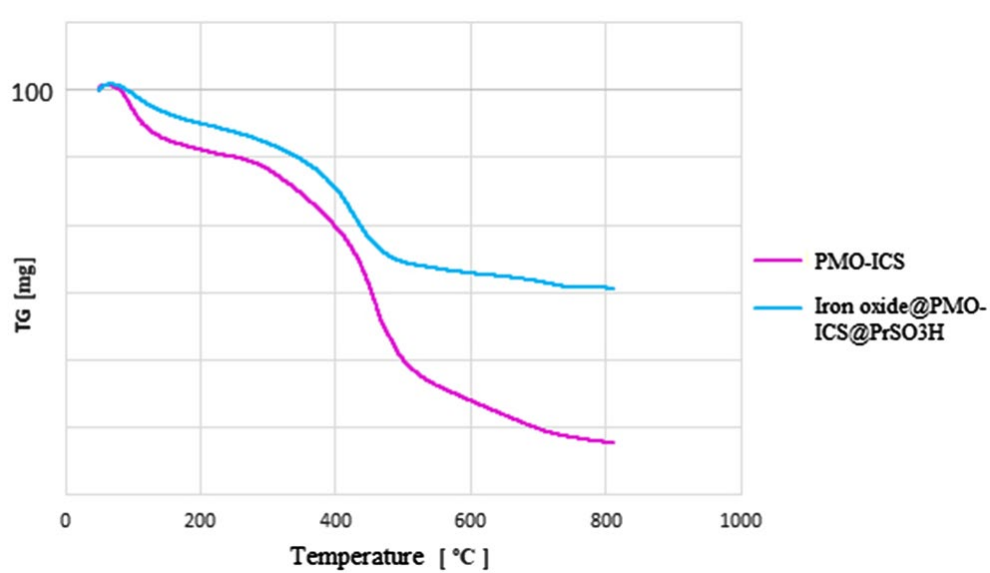
TGA analysis of pure PMO-ICS (purple curve) and Iron oxide@PMO-ICS-PrSO_3_H mesoporous catalysts (**1**, blue curve).

On the other hand, thermogravimetric analysis (TGA) of the Iron oxide@PMO-ICS-PrSO_3_H magnetic catalyst (**1**) demonstrated three weight losses at different temperature ranges. The first one, with 4.96% weight loss between 25 and 100 °C, is corresponded to the removing of water and alcoholic solvents remaining from the extraction process. The second and very small weight loss (1.82%) at 100 to 260 °C region is attributed to the elimination of surfactant template of the synthesis process. Finally, the main weight loss (20.10%) which observed in the range of 260–800 °C, is attributed to the removing of organic functional groups including propylenesulfonic acid and 1,3,5-tris(1,3-propylen) isocyanurate moiety incorporated in the material framework (Fig. 3).

**Figure 4:**
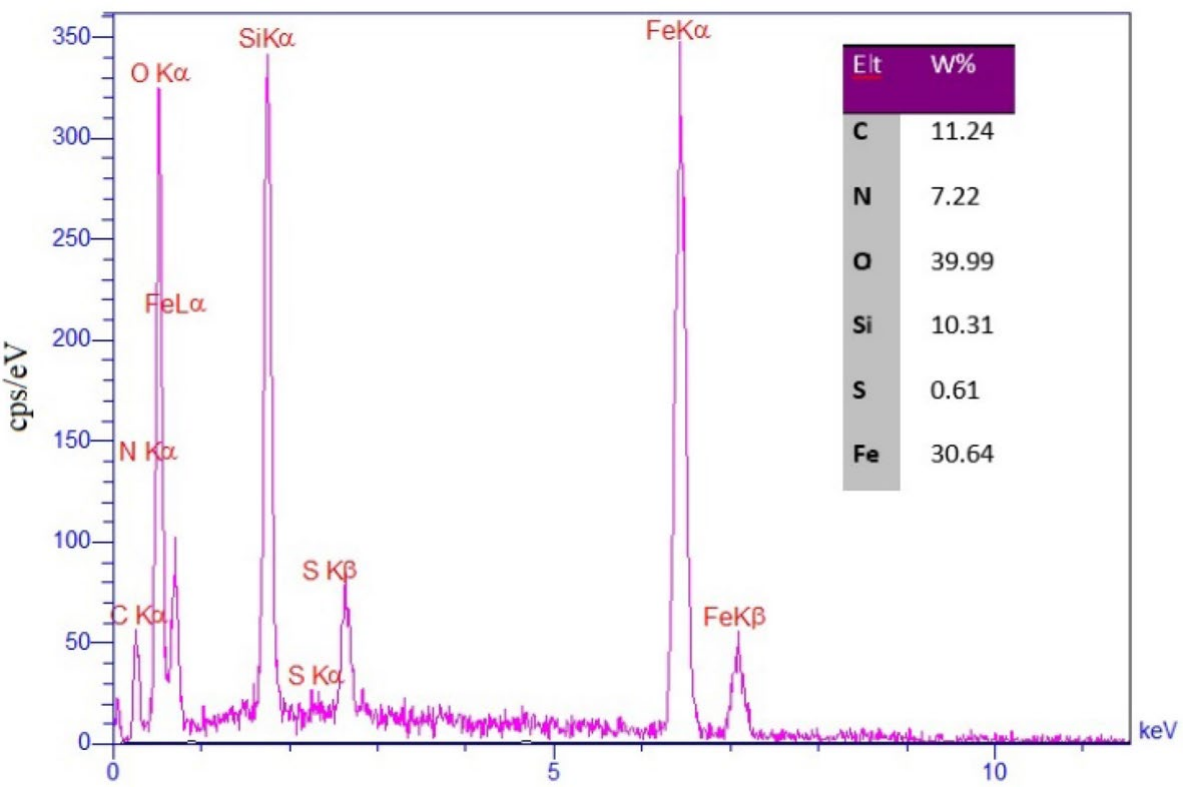
The EDX spectrum of the Iron oxide@PMO-ICS-Pr-SO_3_H mesoporous catalyst (**1**).

Furthermore, the composition of Iron oxide@PMO-ICS-PrSO_3_H mesoporous catalyst (**1**) was characterized by energy-dispersive X-ray (EDX) spectroscopy. As shown in Fig. 4, signals of C, O, N, Fe, Si and S elements (ratios of 11.24: 39.99: 30.64: 7.22: 10.31: 3.64 wt%, respectively) confirm the successful incorporation of expected elements into the structure of Iron oxide@PMO-ICS-PrSO_3_H mesoporous catalyst.

**Figure 5:**
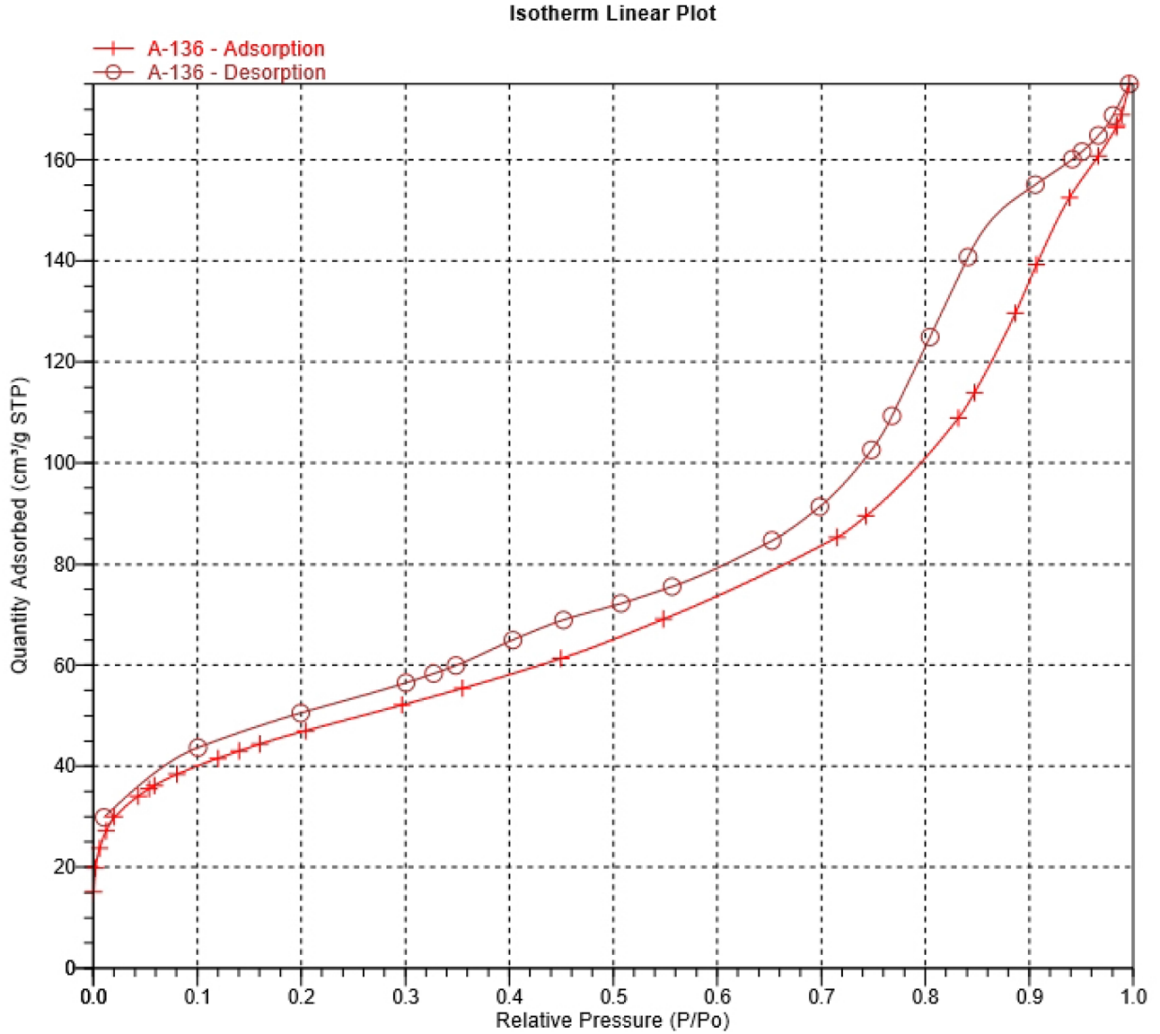
N_2_ adsorption–desorption isotherms of the Iron oxide@PMO-ICS-PrSO_3_H mesoporous materials (**1**).

On the other hand, Fig. 5 shows the N_2_ adsorption–desorption isotherms and pore size distributions (Barrett–Joyner–Halenda, BJH) of the Iron oxide@PMO-ICS-PrSO_3_H mesoporous materials (**1**). The Iron oxide@PMO-ICS-PrSO_3_H itself displays a type IV isotherm, with an H_3_ hysteresis loop. This analysis demonstrated that the BET specific surface area of the mesoporous materials **1** is close to 175.05 m^2^/g and it exhibits BJH average pore diameter and total pore volume equal to 7.41 nm and 0.32 cm^3^/g, respectively.

**Figure 6:**
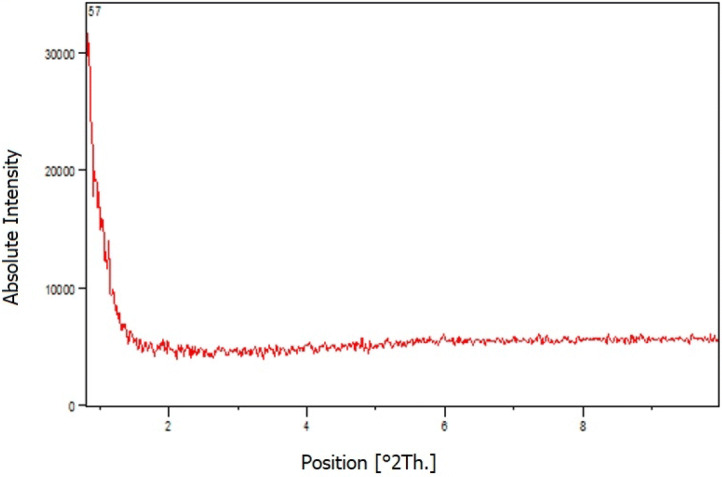
Low-angle XRD patterns of Iron oxide@PMO-ICS-PrSO_3_H solid acid (**1**).

Moreover, the total acidity of Iron oxide@PMO-ICS-PrSO_3_H solid acid (**1**) was calculated through pH analysis of a precisely weighed sample of the material after ion exchange with saturated solution of NaCl. The results demonstrated that the loading of H^+^ on the solid surface is 2.0 mmol.g^−1^. On the other hand, low-angle XRD patterns of Iron oxide@PMO-ICS-PrSO_3_H solid acid (**1**) shows one sharp peak at 2θ =  ~ 0.95 which confirms the presence and preservation of mesoporous framework of the PMO-ICS organosilica as well as its periodicity (Fig. 6).

**Figure 7:**
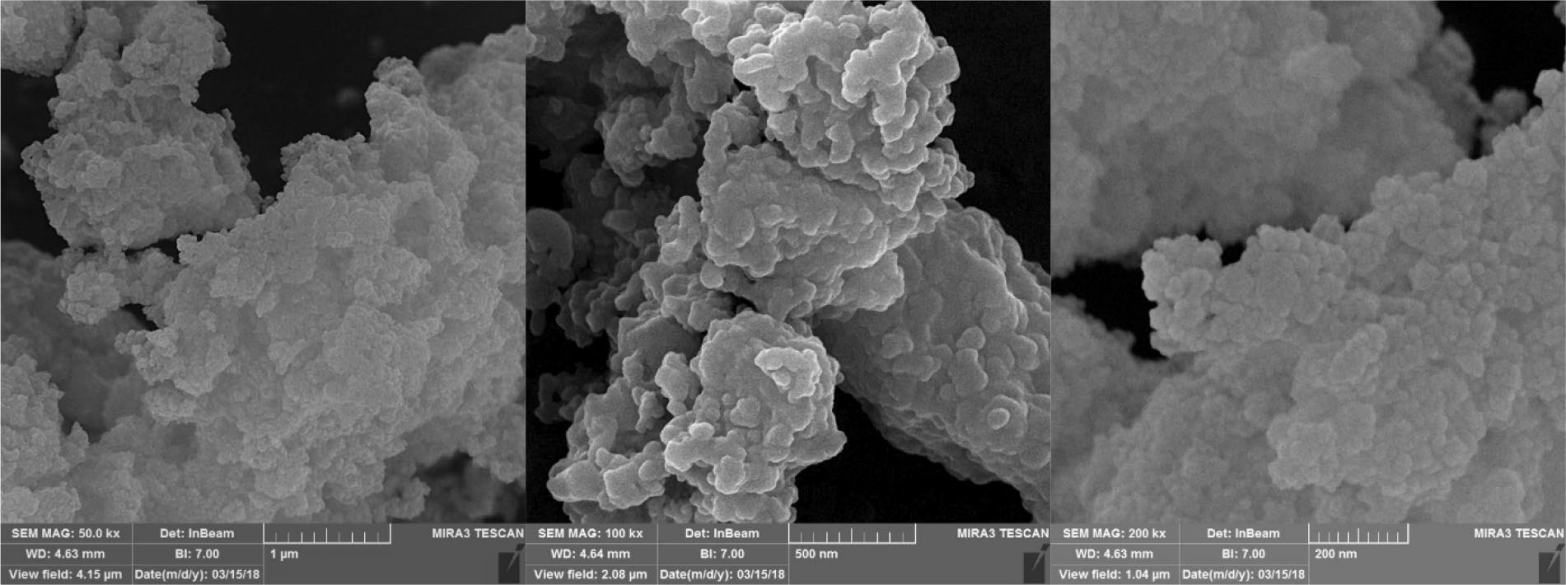
FESEM images of the Iron oxide@PMO-ICS-PrSO_3_H magnetic mesoporous nanocatalyst (**1**).

Furthermore, the morphology of the catalyst (**1**) was characterized by field emission scanning electron microscopy (FESEM). The FESEM images of Iron oxide@PMO-ICS-PrSO_3_H powder (**1**) illustrated well-ordered structure of PMO-ICS and almost uniform distribution of propylenesulfonic acid functional group and iron oxide particles with average particle sizes of about 14–32 nm (Fig. 7).

**Figure 8:**
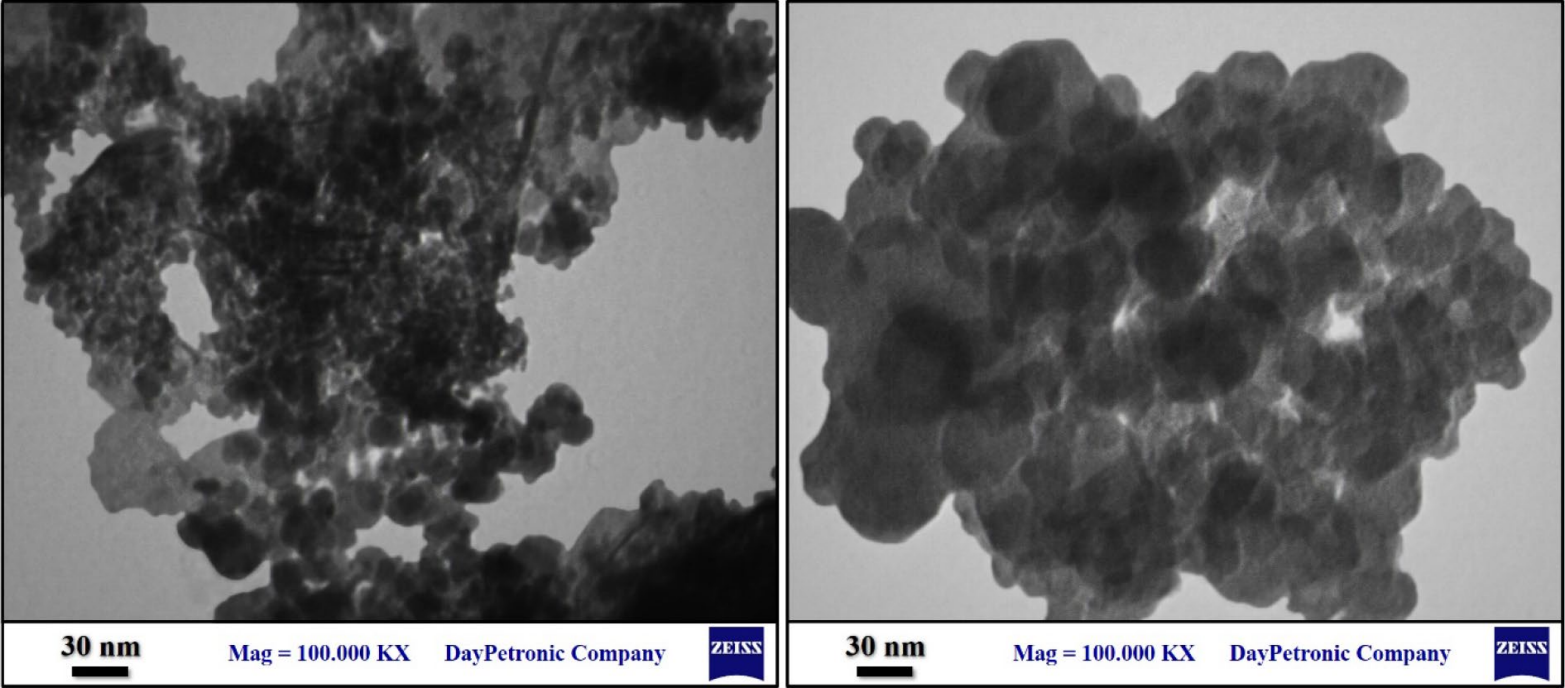
TEM images of Iron oxide@PMO-ICS-PrSO_3_H nanocatalyst (**1**).

Furthermore, TEM images illustrated the structural order and the morphology of Iron oxide@PMO-ICS-PrSO_3_H nanocatalyst (**1**) as well as presence of well distributed iron oxide nanoparticles confined inside of its mesoporous channels **(**Fig. 8).

**Figure 9:**
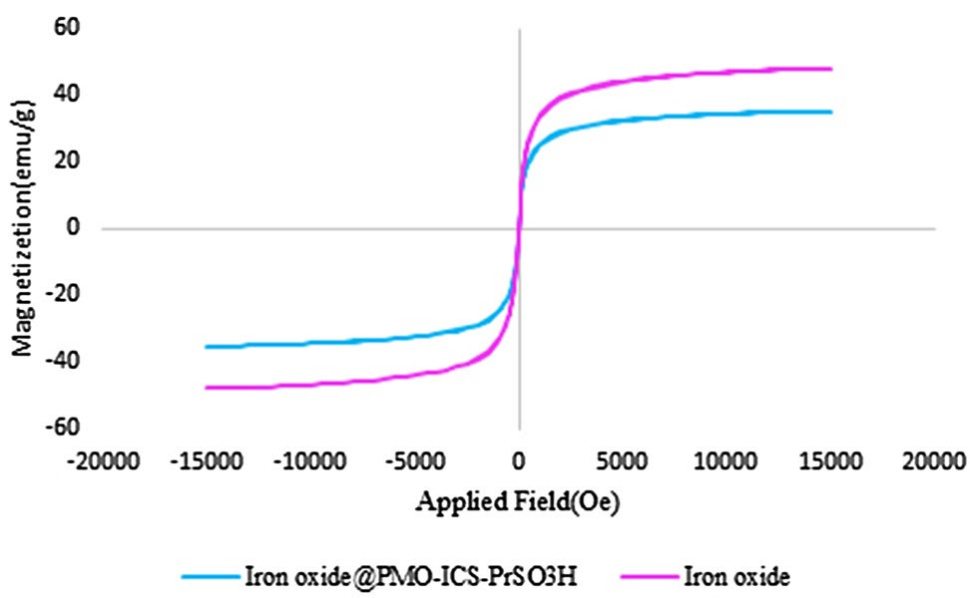
VSM pattern of the iron oxide (purple curve) and the Iron oxide@PMO-ICS-PrSO_3_H mesoporous catalyst (**1**, blue curve).

Also, the saturation magnetic properties of Iron oxide@PMO-ICS-PrSO_3_H mesoporous materials (**1**) were evaluated using VSM technique at room temperature. According to the obtained results shown in Fig. 9, the saturation magnetization of the Iron oxide@PMO-ICS-PrSO_3_H mesoporous materials was determined to be 35 emu/g which is lower than that of the parent superparamagnetic iron oxide (55 emu/g) but is sufficiently high for practical applications^100,101^.

### Investigation of the catalytic activity of the Iron oxide@PMO-PrSO_3_H nanocatalyst (1) for the synthesis of imidazopyrimidine derivatives 6a-g or 7a–g

In this step, the catalytic activity of the Iron oxide@PMO-PrSO_3_H nanocatalyst (**1**) was investigated for the synthesis of imidazopyrimidine derivatives. Therefore, the reaction of 2-aminobenzoimidazole (**2**) and 4-chlorobenzaldehyde (**3a**) with dimedone (**4**) was selected as the model reaction. The obtained results from optimization experiments illustrated that both the catalyst loading and temperature strongly affect the reaction progress which have been summarized in Table 1. Indeed, only a trace amount of the desired product, 12-(4-chlorophenyl)-3,3-dimethyl-3,4,5,12-tetrahydrobenzo[4,5]imidazo[2,1-*b*]quinazolin-1(2*H*)-one (**6a**), was obtained in the absence of Iron oxide@PMO-ICS-PrSO_3_H nanocatalyst (**1**) after 2 h at 80 °C under solvent-free conditions (Table 1, entry 1). To our deligh, the use of Iron oxide@PMO-ICS-PrSO_3_H (**1**) at 7 mg loading enhanced significantly the yield of the desired product **6a** under the same conditions (Table 1, entry 2). Increasing of the catalyst loading to 10 mg (Iron oxide@PMO-ICS-PrSO_3_H, **1**) afforded higher yield of the desired product in shorter reaction time (entry 3). However, higher loading of the solid acid catalyst **1** had no significant impact on the yield and reaction time (entries 4,5). On the other hand, lower yields of the desired product **6a** were obtained when the model reaction was investigated at lower temperatures under same catalyst loading or solvent-free conditions at 80 °C (Table 1, entries 6–7). Furthermore, 12-(4-chlorophenyl)-3,3-dimethyl-1,2,3,4,5,12-hexahydrobenzo[4,5]imidazo[2,1-*b*]quinazolin-1-one (**6a**) was obtained in lower yields when the model reaction was investigated using 10 mg loading of Iron oxide@PMO-ICS-PrSO_3_H (**1)** in other solvents such as EtOH/H_2_O or THF under reflux conditions (Table 1, entries 8–9). Moreover, lower yields of the desired product **6a** was obtained in the presence of Iron oxide@PMO-ICS, Iron oxide@PMO-ICS-PrSH, iron oxide, or pure PMO-ICS under similar reaction conditions (10 mg catalyst loading, solvent-free conditions, 80 °C**,** Table 1, entries 10–13). These findings indicate that the catalytic activity of Iron oxide@PMO-ICS-PrSO_3_H is mainly related to the existence of significant synergic effect of sulfonic acid groups (–SO_3_H) along with iron oxide in this mesoporous catalyst. Furthermore, the Sheldon test was performed to show the heterogeneous nature of the magnetic catalyst **1** and verify possible leaching of the propylsulfonic acid groups to the reaction mixture^102^. Thus, the catalyst **1** was isolated from the reaction mixture by an external magnet after 5 min heating at 80 °C (10 mg catalyst loading) and the remaining mixture was heated for further 10 min. Indeed, only 57% of the desired product **6a** was isolated.

**Table 1:** Optimization of the conditions for the model reaction in the synthesis of imidazopyrimidine derivative **6a.**
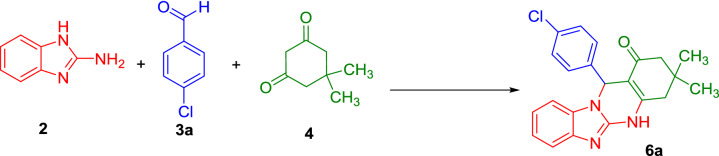

Entry	Catalyst	Catalyst loading (mg)	Solvent		Temp. (°C)	Time (min)	Yield^a^ (%)
1	-	-	Solvent-Free		80	120	Trace
2	Iron oxide@PMO-PrSO_3_H	7	Solvent-Free		80	15	92
3	Iron oxide@PMO-PrSO_3_H	10	Solvent-Free		80	10	95
4	Iron oxide@PMO-PrSO_3_H	15	Solvent-Free		80	10	96
5	Iron oxide@PMO-PrSO_3_H	20	Solvent-Free		80	7	97
6	Iron oxide@PMO-PrSO_3_H	10	Solvent-Free		70	15	93
7	Iron oxide@PMO-PrSO_3_H	10	Solvent-Free		60	20	93
8	Iron oxide@PMO-PrSO_3_H	10	EtOH/H_2_O		60	25	82
9	Iron oxide@PMO-PrSO_3_H	10	THF		Reflux	30	75
10	Iron oxide@PMO	10	Solvent-Free		80	35	65
11	Iron oxide@PMO-PrSH	10	Solvent-Free		80	40	36
12	Iron oxide	10	Solvent-Free		80	70	28
13	PMO-ICS	10	Solvent-Free		80	50	46

Reaction conditions: 2-aminobenzoimidazole (**2**, 1 mmol) and 4-chlorobenzaldehyde (**3a,** 1 mmol) and dimedone (**4**, 1 mmol) under solvent-free conditions (unless solvent indicated, 2 mL).

^a^Isolated yields.

In the next step, the activity of the Iron oxide@PMO-ICS-PrSO_3_H catalyst **1** in the synthesis of imidazopyrimidines derivatives was further investigated to other aromatic aldehydes **3b–h** or malononitrile C–H acid **5** using optimized conditions (10 mg Iron oxide@PMO-ICS-PrSO_3_H loading under solvent-free conditions at 80 °C). Indeed, different derivatives of imidazopyrimidine were prepared in high to excellent yields via the condensation of 2-aminobenzimidazole (**2**), aromatic aldehydes **3a-g**, dimedone (**4**) or malononitrile (**5**) under optimal reaction conditions. As data in Table 2 show, various aromatic carbocyclic or heterocyclic aldehydes including both electron-withdrawing and electron-donating group were involved in the optimal reaction conditions to afford the desired products **6–7** in high to excellent yields (Table 2). In all studied cases, the reaction proceeded smoothly and the desired products were obtained without remaining any intermediates after reaction times indicated in Table 2. The obtained products were identified by the comparison of their spectral data and melting points with those reported for the valid samples.

**Table 2:** Synthesis of the imidazopyrimidine derivatives **6a–g** and **7a–g** in the presence of the Iron oxide@PMO-ICS-PrSO_3_H nanocatalyt (**1**).
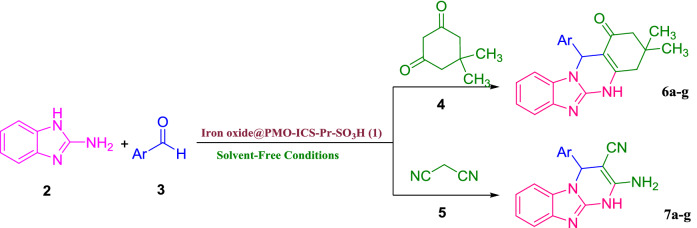

Entry	Aldehyde **3**	C − H acid **4–5**	Product **6–7**	Time (min)	Yield^a^ (%)	M.P [Lit.]
1	4-Chlorobenzaldehyde (**3a**)	**4**		15	95	336–339 ^80^
2	2-Chlorobenzaldehyde (**3b**)	**4**		18	92	352–355 ^80^
3	4-Methylbenzaldehyde (**3c**)	**4**		18	92	325–328 ^103^
4	4-Methoxybenzaldehyde (**3d**)	**4**		20	90	385–388 ^104^
5	4-Nitrobenzaldehyde (**3e**)	**4**		15	95	374–378 ^105^
6	Benzaldehyde (**3f**)	**4**		25	88	308–310 ^106^
7	4-Pyridinbenzaldehyde (**3 g**)	**4**		14	95	298–300 ^92^
8	4-Chlorobenzaldehyde (**3a**)	**5**		10	96	234–237 ^80^
9	2-Chlorobenzaldehyde (**3b**)	**5**		15	93	236–238 ^92^
10	4-Methylbenzaldehyde (**3c**)	**5**		14	92	189–201 ^80^
11	4-Methoxybenzaldehyde (**3d**)	**5**		18	95	231–234 ^80^
12	4-Nitrobenzaldehyde (**3e**)	**5**		12	94	345–348 ^92^
13	Benzaldehyde (**3f**)	**5**		20	90	232–235 ^107^
14	3-Nitrobenzaldehyde (**3 h**)	**5**		18	93	235–237 ^107^

Reaction conditions: 2-aminobenzoimidazole (**2**, 1 mmol) and 4-chlorobenzaldehyde (**3a**, 1 mmol) and dimedone (**4**, 1 mmol) in the presence of Iron oxide@PMO-ICS-PrSO_3_H (**1**, 10 mg).

^a^Isolated yields.

**Scheme 2:**
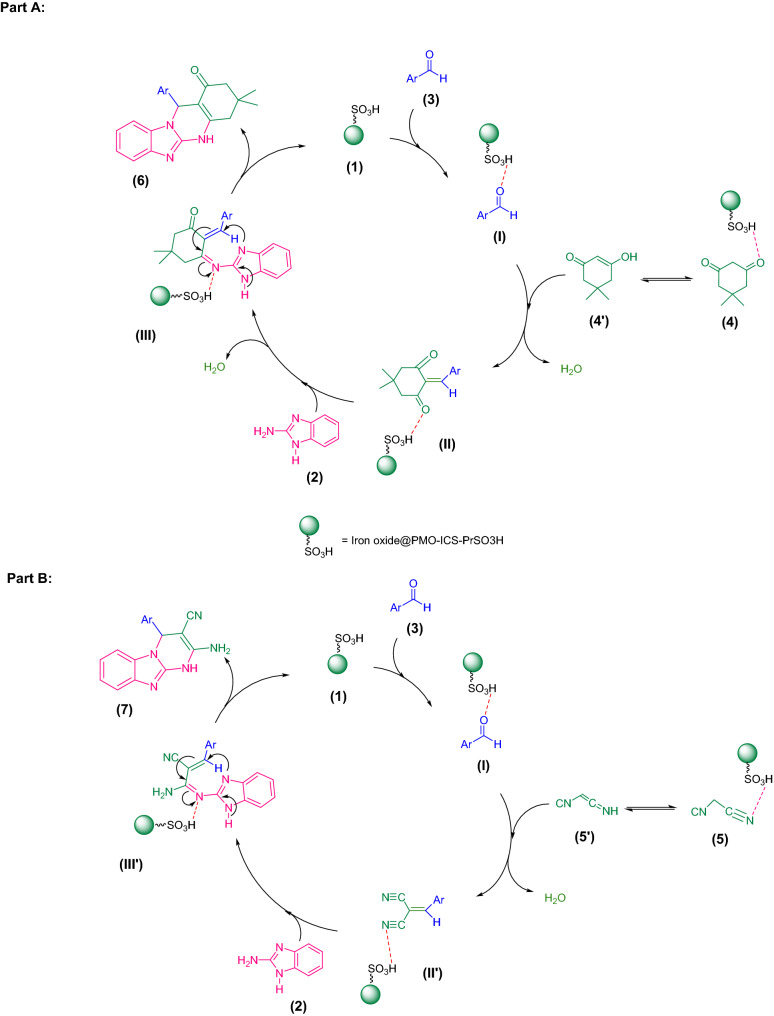
A plausible mechanism for the synthesis of imidazopyrimidine derivatives **6a–g** (Part A) and **7a–g** (Part B) in the presence of the Iron oxide@PMO-ICS-PrSO_3_H nanocatalyst (**1**).

According to the obtained results, a plausible mechanism for the synthesis of imidazopyrimidine derivatives **6a–g** and **7a–g** catalyzed by the Iron oxide@PMO-ICS-PrSO_3_H nanocatalyst (**1**) is outlined in Scheme 2. At the first step, aldehydes **3** can be activated by the Iron oxide@PMO-ICS-PrSO_3_H magnetic solid acid mainly through -PrSO_3_H groups to afford the Knoevenagel condensation product of aldehydes **3** and dimedone (**4**) or malononitrile (**5**) C–H acid as intermediates (II) or (II’), respectively. Then, condensation of these intermediates with 2-aminobenzoimidazole (**2**) produces Michael acceptor intermediates (III) or (III’), respectively, in the presence of Iron oxide@PMO-ICS-PrSO_3_H solid acid (**1**). Finally, the activated intermediates (III) or (III’) by the mesoporous Iron oxide@PMO-ICS-PrSO_3_H catalyst (**1**) involve in the intramolecular Michael addition and subsequent tautomerization to afford tetracyclic or tricyclic imidazopyrimidine derivatives **6a–g** and **7a–g,** respectively.

### The recyclability of the Iron oxide@PMO-ICS-PrSO_3_H catalyst (1) in the synthesis of imidazopyrimidine derivatives

**Figure 10:**
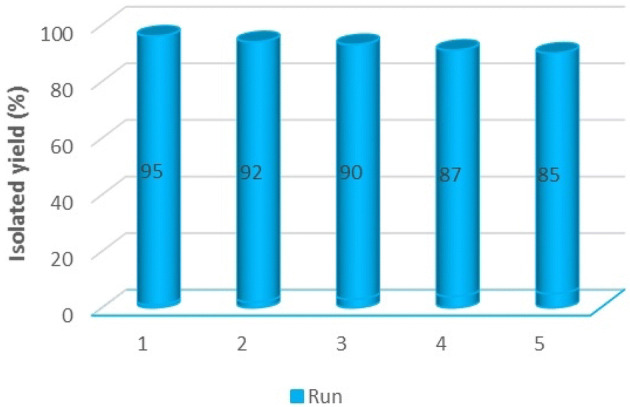
Investigation of the feasibility for reusing of Iron oxide@PMO-ICS-PrSO_3_H magnetic mesoporous catalyst (**1**) in the synthesis of 12-(4-chlorophenyl)-3,3-dimethyl-1,2,3,4,5,12-hexahydrobenzo[4,5]imidazo[2,1-*b*]quinazolin-1-one (**6a**).

In this part of our study, the recyclability of the Iron oxide@PMO-ICS-PrSO_3_H catalyst (**1**) in the model reaction was investigated under optimized conditions. The catalyst was easily recovered from the reaction mixture by an external magnet in each run, then washed with water and EtOH and finally dried at 100 °C for 2 h before next run (Fig. 10). During the recycling experiments with the reactants of model reaction under the same reaction conditions, no significant change in the activity of the catalyst (**1**) was observed for at least five successive runs, which clearly demonstrates the stability of the catalyst in synthesis of imidazopyrimidine derivatives under optimized conditions.

Finally, to demonstrate the merits of the newly developed solid acid catalyst **1** in the synthesis of imidazopyrimidine derivatives, the present protocol has been compared with other methods and published reports. Table 3 summarizes these data.

**Table 3:** Comparison of the catalytic efficiency of the Iron oxide@PMO-ICS-PrSO_3_H solid acid (**1**) with other catalytic systems for the synthesis of **6a**.

Entry	Catalyst	Catalyst loading	Solvent	Temp (°C)	Time (min)	Yield (%)	References
1	Fe_3_O_4_@GO	65 mg	EtOH	Reflux	125	94	^24^
2	[PVP-SO_3_H]HSO_4_	25 mg	Solvent-Free	90	14	96	^43^
3	Fe_3_O_4_@IM	30 mg	EtOH	78	15	95	^10^
4	Nano-WO_3_-SO_3_H	19 mg	Solvent-Free	100	14	91	^5^
5	Fe_3_O_4_@SiO_2_–ZrCl_2_-MNP	25 mg	Solvent-Free	100	8	95	^32^
6	Iron oxide@PMO-ICS-Pr-SO_3_H	10 mg	Solvent-Free	80	10	95	This Work

## Experimental section

### General information

All chemicals and reagents were supplied by Aldrich or Merck chemical companies. Benzaldehyde was used as a fresh distilled sample and other aldehydes were used without further purification. Commercial Merck silica gel 60 coated with flourescent indicator F254 on aluminium plates were used in thin layer chromatography (TLC) experiments to monitor the progress of reactions. Transmission electron microscope, TEM (Zeiss EM10C, Germany) was used to obtain TEM images. A MIRA3 instrument of TESCAN Company, Czech Republic was used to obtain field emission scanning electron microscopy (FESEM) images. XRD patterns were obtained using an X-ray powder X'Pert Pro PANalytical diffractometer with CuKα radiation source. Thermal gravimetric analysis (TGA) was accomplished by means of a Bahr company STA 504 instrument. An ASAP 2020 micromeritics equipment was used to determine the BET specific surface area of the catalyst. FTIR spectra were obtained using KBr disks on a Shimadzu FT IR-8400S spectrometer. Melting points were determined using a digital melting point Electrothermal 9,100 apparatus and are uncorrected. ^1^H NMR (500 MHz) spectra were obtained using a Bruker DRX-500 AVANCE spectrometer in DMSO at ambient temperature. VSM analysis was performed using a Lakeshore 7,410 series instrument.

### General procedure for the preparation of magnetic isocyanurate-based periodic mesoporous organosilica (Iron oxide@PMO-ICS) nanomaterials (B)

Isocyanurate-based periodic mesoporous organosilica (PMO-ICS) nanomaterials (A) were prepared according to the procedure described in our previous publications^34,98^. After that, PMO-ICS (A, 2.0 g) was dispersed in toluene (20 mL) and stirred for 20 min at room temperature. Then, FeCl_2_.4H_2_O (2.0 g) and FeCl_3_.6H_2_O (4.0 g) were added to the obtained mixture under nitrogen atmosphere. The reaction mixture was then heated in an oil bath at 80 °C for 1 h. Next, aqueous NH_3_ (25% w/v, 20 mL) solution was added dropwise to the reaction mixture over 30 min and the reaction allowed to proceed further for 1 h at 80 °C. Then the obtained solid was washed with deionized H_2_O/EtOH (50:50 v/v, 40 mL) and dried at 100 °C for 1 h.

### General procedure for the preparation of magnetic isocyanorate-based propylsulfonic acid periodic mesoporous organosilica (Iron oxide@PMO-ICS-PrSO_3_H) nanomaterials (1)

Iron oxide@PMO-ICS (**B**, 2.0 g) was dispersed in toluene (10 mL). Then, 0.4 mL of the 3-[(trimethoxysilyl) propyl] thiol was slowly added to the mixture and stirred at room temperature for 24 h to afford Iron oxide@PMO-ICS-PrSH (**C**). The resulting solid was filtered, washed by distilled water and dried under vacuum for 1 h. Finally, 1.0 g of Iron oxide@PMO-ICS-PrSH (**C**) was dispersed in deionized H_2_O (4 mL) and H_2_O_2_ (6 mL) was slowly added to the above mixture stirred at room temperature for 24 h. The obtained black solid (Iron oxide@PMO-ICS-PrSO_3_H, **1**) was filtered off and washed with deionized water twice (15 mL) and then dried at 100 °C for 2 h.

### General procedure for the synthesis of imidazopyrimidine derivatives 6/7 a-g catalyzed by Iron oxide@PMO-ICS-PrSO_3_H nanomaterials (1)

Iron oxide@PMO-ICS-PrSO_3_H (**1**, 10 mg) was added to a mixture of 2-aminobenzoimidazole (**2**, 1 mmol, 0.133 mg), aromatic aldehyde (**3**, 1 mmol), and dimedone or malononitrile (**4-5**, 1 mmol). The obtained reaction mixture was stirred under solvent-free conditions at 80 °C for the proper times indicated in Table 2. The progress of the reaction was monitored by TLC (EtOAc: n-hexane, 1:3). After completion of the reaction, DMF (2 mL) was added and the reaction mixture was heated to dissolve organic materials. The magnetic nanocatalyst **1** was then collected by an external magnet. After that, distilled water (5 mL) was added to the DMF solution and the obtained precipitate was filtered off and washed using n-hexane (2 mL) to afford pure products. The obtained powders were then dried in an oven at 80 °C for 1 h.

## Conclusions

In summary, the novel and thermally stable magnetic isocyanorate-based propylsulfonic acid periodic mesoporous organosilica (Iron oxide@PMO-ICS-PrSO_3_H) was prepared for the first time. The Iron oxide@PMO-ICS-PrSO_3_H solid acid was used for highly efficient, facile, and green and sustainable synthesis of 12-phenyl-3,3-dimethyl-3,4,5,12-tetrahydrobenzo[4,5]imidazo[1,2-*b*]quinazolin-1(2*H*)-one or 2-amino-4-phenyl-1,4-dihydrobenzo[4,5]imidazo[1,2-*a*]pyrimidine-3-carbonitrile derivatives in a one-pot and three-component protocol through condensation of aldehydes, dimedone/malononitrile, and 2-aminobenzimidazole under solvent-free conditions. This methodology offers outstanding advantages including (i) high to excellent yields in shorter reaction times, (ii) low catalyst loading and cost and (iii) simple work-up procedure, fast separation of the catalyst, and catalyst recyclability.
